# Daidzein Alleviates Hypothalamic-Pituitary-Adrenal Axis Hyperactivity, Ameliorates Depression-Like Behavior, and Partly Rectifies Circulating Cytokine Imbalance in Two Rodent Models of Depression

**DOI:** 10.3389/fnbeh.2021.671864

**Published:** 2021-10-18

**Authors:** Long Chen, Xiaokun Wang, Yunpeng Zhang, Hequan Zhong, Cuiting Wang, Pengfei Gao, Bing Li

**Affiliations:** ^1^Department of Neurology, Jinshan Hospital, Fudan University, Shanghai, China; ^2^Research Center for Clinical Medicine, Jinshan Hospital Affiliated to Fudan University, Shanghai, China; ^3^Department of Traditional Chinese Medicine, Jinshan Hospital Affiliated to Fudan University, Shanghai, China

**Keywords:** depression, daidzein, HPA axis, learned helpless, chronic mild stress, inflammatory cytokines

## Abstract

Depression is one very common mental health disorder which can cause morbidity and mortality if not addressed. Recent studies have provided strong evidence that depression may be accompanied by immune activation, secondary inflammatory reaction, and hyperactivity of the Hypothalamic Pituitary Adrenal (HPA) axis. It is well-known that it takes at least 2 weeks for conventional antidepressants, especially SSRIs (Selective serotonin reuptake inhibitors) to produce effects. To better understand the mechanism of antidepressant effects on depression and subsequently further elucidate the pathogenesis of depression, we selected phytestrogen daidzein (DD) to observe its effects on the depression-like and anxiety-like behavior in two different rodent models of depression which were induced by learned helplessness and chronic mild stress (CMS) and then simultaneous evaluation of the depression-like behavior, the activity of HPA axis, and circulatory cytokines. Our results showed that daidzein attenuated depression-like behaviors through alleviating HPA axis hyperactivity, decreasing the levels of stress-related hormones, and partly rectifying some inflammatory cytokines imbalance in both the rodent models of depression.

## Introduction

Mood and anxiety disorders have become one of the leading causes of disabilities and burdens in modern society with respect to direct medical costs and productivity loss (Vollbehr et al., 2018). As a major mental disease, depression affects approximately 12%–17% of the global population, at some point in their lives (Lopez-Torres, 2019). It is characterized by multifactorial clusters of symptoms and etiologies, including feelings of despair, helplessness, and avoidance/withdrawal from social situations (Alharbi et al., 2018).

The pathogenesis of anxiety disorders is unclear. There is a close relationship between serotonin and anxiety disorders. Selective serotonin reuptake inhibitors (SSRIs) are the most commonly prescribed class of antidepressants, although their therapeutic effects do not emerge until several weeks later (Harmer and Cowen, 2013). Less than one-third of patients with major depression attain remission with an initial antidepressant trial, which means that there may be other pathogeneses of anxiety disorder. Therefore, it is necessary to further explore the pathogenesis of anxiety and identify new antianxiety drugs.

In addition to deficits in the serotonin system, hyperactivity of the hypothalamic-pituitary-adrenal (HPA) axis was also considered to be associated with the etiology of depression, which is supported by the findings that many depressive patients exhibit various abnormalities in HPA regulation (Lu et al., 2016), and chronic over-activation of the HPA axis also causes injuries to the brain and body *via* the toxic effects of elevated cortisol levels (Gerritsen et al., 2019). Moreover, normalization of HPA axis activity in patients with depressive disorders has shown improved clinical outcomes (Min et al., 2012). In some animal models of depression, antidepressants, anxiolytics, and mood stabilizers have been shown to reduce the overall responsiveness of the HPA axis (Fernandez et al., 2013; Valvassori et al., 2017; Fischer et al., 2019). Hence, it is inferred that aberrant activation of the HPA axis may play a possible role in the pathophysiology of depression. Antidepressant treatment may be affected by correcting the abnormal activity of the HPA axis.

In addition, recent studies have provided solid evidence that depression may be accompanied by immune activation, which is expressed as an increase in pro-inflammatory cytokines at the peripheral (Stark et al., 2001; Quan et al., 2003; Bermudez, 2012) and central levels (Wohleb et al., 2011). Meanwhile, antidepressants have been shown to suppress the production of pro-inflammatory cytokines (Mutlu et al., 2012) while stimulating the production of interleukin (IL)-10, an anti-inflammatory cytokine (Roque et al., 2009). Conversely, immunological activation has been found to induce depression-like symptoms in both humans and animals (Kubera et al., 2000; Zhao et al., 2019), and chronic immune activation or inflammatory processes seem to be particularly evident in treatment-resistant depression (TRD; Sluzewska et al., 1996; Sluzewska, 1999). It has also been hypothesized that hypercortisolaemia observed in patients experiencing major depression may be induced by pro-inflammatory cytokines (Leonard, 2014). Therefore, based on the evidence mentioned above, a causal relationship between the activation of the immune system and the development of depression has recently been proposed; moreover, the alteration in pro-and anti-inflammatory cytokines may be regarded as a biomarker of depression (Aleem and Tohid, 2018; Levada and Troyan, 2018) which may parallel the development of depression (Kim et al., 2018).

Taken together, we presumed that the effects of antidepressants were directly related to rectifying the aberrant alterations of the neuro-immune-endocrine system in depressive patients and animal models of depression, which lay the foundation for our subsequent research.

Estrogen is a female hormone that plays a key role in the regulation of reproductive behavior and control of the neuroendocrine system in both males and females (Baghel and Srivastava, 2020). In adults, generalized anxiety disorder (GAD) is two times more common in women than in men (Hidalgo and Sheehan, 2012). This phenomenon indicates that there is a strong link between estrogen and anxiety. In humans, it is known that high and constant levels of estrogen exert anxiolytic effects, whereas low and fluctuating levels promote dysphoric mood and anxiety (Findikli et al., 2016). Similar effects of estrogen on affective behavior have been observed in animal models. In rodents, ovariectomy which mimics estrogen deficiency can induce a reliable increase in depression and anxiety-like behavior. Replacement with estrogen ameliorates this behavior in these animals (Kiss et al., 2012; Xu et al., 2015). Soy isoflavones are non-steroidal compounds found in plants, with a similar chemical structure as that of 17-β-estradiol and are thus considered to be phytoestrogens. Isoflavones can mimic the binding of estrogens to ERs, exerting estrogenic effects on target organs (Vitale et al., 2013; Zaheer and Humayoun Akhtar, 2017) and exhibit various biological activities. Daidzein (DD), an abundant isoflavone present in soy, is unique as it can be further metabolized into equol, a compound with greater estrogenic activity than other isoflavones, exhibiting many kinds of bioactivities, such as anti-oxidation (Zhihua et al., 2010) and endocrine system regulation (Zhang et al., 2018). Some studies have found that daidzein has sex-related effects. For example, some epidemiologic studies have speculated that dietary phytoestrogens, such as daidzein, may play a role in the reduced risk of prostate cancer in men. Meanwhile, soybean phytoestrogens, such as genistein and daidzein, have become popular alternatives for women undergoing treatment for menopausal symptoms. However, most recently, researchers have noticed that the long–term consumption of daidzein may produce significant effects on locomotor activity, mood, and social behavior without significant effects on learning and memory. However, its mechanism of action remains unclear. There are some reports that daidzein may regulate inflammatory response and cortisol secretion (Meng et al., 2017; Das et al., 2018; Zeng et al., 2019). Svetlana et al. revealed that histological and morphofunctional parameters of the HPA system were sensitive to daidzein treatment in adult rats (Trifunović et al., 2018). They found that daidzein inhibited adrenocorticotropic hormone (ACTH)-induced cortisol production in fetal and postnatal cells and reduced cAMP-stimulated cortisol release from H295 cells. In addition, Caceres found that daidzein could cause decreased cortisol secretion during puberty in male Wistar rats (Caceres et al., 2014).

Thus, we selected daidzein to observe its effects on depression-like and anxiety-like behaviors in two different rodent models of depression *via* assessment of the activity of the HPA axis and detection of the related circulatory inflammatory cytokines.

## Materials and Methods

### Animals

The experiments were performed on male Wistar rats weighing 200–220 g and male C57BL/J6 mice weighing 23–27 g [from the Experimental Animal Centre, Chinese Academy of Science (Shanghai, China)]. Male Wistar rats were used for the establishment of the learned helpless paradigm as a sub-chronic model and male C57BL/J6 mice were used for establishing chronic-mild-stress-paradigm. The animals were housed in an air-conditioned room (22°C) with a 12:12 h light–dark cycle with free access to food and water. All animal experiments were approved by the Ethics Committee of the Animal Care of Jinshan Hospital. All experimental procedures were performed in accordance with the National Institutes of Health Guide for the Care and Use of Laboratory Animals.

#### Drugs and Solvents

Daidzein was purchased from Sigma Chemical Co. (St. Louis, MO, USA). Daidzein was dissolved in a minimal volume of absolute ethanol and mixed with sterile olive oil. A mixture of anhydrous ethanol and olive oil (in a 1:9 ratio) was used as the vehicle.

#### Learned Helpless Paradigm of Rats

**Figure 1:**
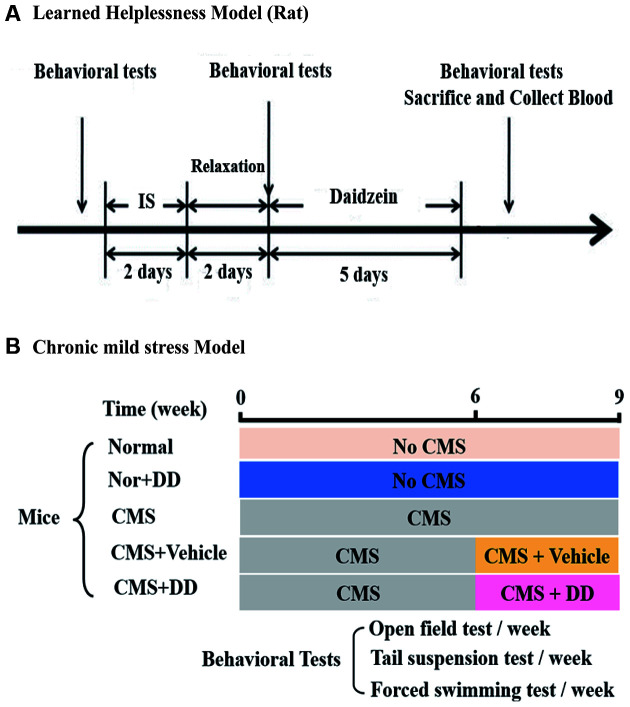
The part of experimental design. **(A)** Behavioral tests were performed before inescapable shock and repeated after inescapable shock on two consecutive days followed by 2 days of rest. After that, the rats fitting a “helpless” pattern were randomly divided into three groups: learned helpless (LH), LH + vehicle, and LH + DD groups, respectively. After the last behavioral test, the animals were sacrificed, and their blood was collected. **(B)** The chronic mild stress (CMS) model was established over 9 weeks. In the 6th week, some of the mice were sacrificed and their blood was collected while some were given daidzein (DD) and others were treated with vehicle, separately. During this process, all mice were still under CMS stimulation. Behavioral tests were performed once a week for the mice. After the last behavioral test, all the mice were sacrificed and their blood was collected.

The experimental design of this part is shown in Figure 1A. The rats were randomly divided into five groups: normal group (*n* = 8), normal + daidzein (DD) group (*n* = 8), learned helpless (LH) model group (*n* = 8), LH + vehicle group (*n* = 8), and LH + DD group (*n* = 8). The learned helpless procedure was performed as described previously (Shirayama and Hashimoto, 2018). Briefly, it included three stages: helplessness induction, active avoidance test, and behavioral tests.

#### Helplessness Induction (Inescapable Foot-Shock Preconditioning)

For two consecutive days, rats were placed in Plexiglas chambers (length × width × height, 45 × 45 × 45 cm) with a stainless-steel grid floor consisting of rods spaced 1 cm apart (Shanghai Mobile Datum Information Technology Company, Shanghai, China) and exposed to 60 s in escapable electric foot shocks (0.8 mA) that were randomly delivered through the steel grid for 15 s every 60 ± 15 s. A constant current shocker was used to deliver the scrambled foot shocks.

##### Active Avoidance Test

Escape/avoidance behavior was evaluated in a shuttle box (60 × 21 × 30 cm; Shanghai Mobile Datum Information Technology Company, Shanghai, China), which consisted of black Plexiglas walls with a stainless-steel grid floor formed by rods spaced 1 cm apart. A black Plexiglas divider with a hole (7 × 7 cm) was placed in the middle of the box to divide it into two equal-sized compartments. For each trial, an acoustic signal (85 dB, conditioned stimuli) was given within the first 3 s prior to the escapable foot-shock administration; if no response occurred within this 3-s-period, a 0.8 mA escapable shock (30 s duration) was subsequently applied. A total of 30 trials were performed, and the number of escape failures during the shock application was recorded. Animals that failed to escape between the 10th and 30th trials were considered “helpless,” whereas those who failed to escape less than five times were considered “Non-helpless”.

##### Behavioral Tests

The depression-like and anxiety-like behaviors of the rats were detected by forced swimming test (FST), open field test (OFT), and elevated plus maze test in addition to the shuttle-box test before and after the inescapable shock and after medication (Figure 1A).

##### Forced Swimming Test

The FST was performed according to a previous description (Hansen et al., 1997) as follows: the rats were forced to swim individually in a cylindrical glass container (40 cm in height and 18 cm in diameter) with tap water (25 ± 1°C) to a depth adjusted for the weight of the individual animal so that its hind paws could just touch the bottom of the container for 5 min. The test sessions were recorded, and the times of immobility and struggling were scored by an observer who was blinded to the grouping of animals. The behavioral test was subsequently scored with reference to the criteria established in a previous study (Gil and Armario, 1998). Immobility was recognized when the rats remained floating passively in the water in a slightly hunched, yet upright position with their heads just above the surface, while struggling was recognized when the rats made one or a few of the following active movements: with their forepaws usually directed against the walls, in and out of the water, or sweeping their heads around or raising their heads above the water while their forelimbs were against the wall.

##### Open Field Test

In this paradigm, each rat was placed in the center of an open field (100 × 100 × 40 cm) constructed of Plexiglas and allowed to explore the field for 5 min. The activity of each rat was monitored using a video tracking system (Shanghai Mobile Datum Information Technology Company, Shanghai, China). The total distance and the number of rearing in the open field were measured.

##### Elevated Plus Maze Test

The maze was built on an elevation 50 cm away from the floor with four 50 × 10 cm arms and a 10 × 10 cm center platform. The two arms were enclosed by 40 cm walls, and the other two arms remained open. The rats were placed in the center of the maze facing an open arm and allowed to explore the maze for 5 min. The activities of the rats were videotaped (Shanghai Mobile Datum Information Technology Company, Shanghai, China), and entering an arm was recognized when all four paws crossed over a particular arm.

#### Chronic Mild Stress Paradigm

The experimental design of this part is shown in Figure 1B. Mice were randomly assigned to five groups: normal group (*n* = 8), normal + DD group (*n* = 8), chronic mild stress (CMS) model group (*n* = 8), CMS + vehicle group (*n* = 8), and CMS + DD group (*n* = 8). In the chronic model experiment, to continuously observe the anxious behavior of the animals and ensure the stability of the chronic mild stress model, we conducted a weekly behavioral test, which not only ensured the observation of animal behavior but also avoided the establishment of adaptive learning and conditioned reflex caused by repeated tests. The procedure to produce chronic mild stress was modified based on a previous report (Mao et al., 2009).

##### Chronic Mild Stress Procedure

In the CMS procedure, mice were chronically subjected to a random sequence of the following unpredictable mild stressors: 60-min cage rotation, reversal of the light/dark cycle, 24-h food deprivation, 24-h water deprivation, 5-min hot environment (45°C), tube restraint (1 h), and soiled cage (200 ml water in 100 g sawdust bedding) for 9 weeks. Normal animals were left undisturbed in their cages.

#### Behavioral Tests

##### Forced Swimming Test

The mouse FST was performed according to a previously described method with slight modifications (Porsolt et al., 1977) as follows: the mice were forced to swim in a transparent glass vessel (25 cm high, 14 cm in diameter) filled with 10-cm deep water at 24–26°C. The water was changed after each trial. The total duration of immobility was measured during the last 5 min of a single 6-min test session by an unbiased observer who was blinded to the grouping of mice. Immobility was defined as floating and making only the necessary movements to keep the head above the water.

###### Tail Suspension Test (TST)

The mouse TST was performed as described previously (Steru et al., 1985). Briefly, the mice were suspended 50 cm above the floor with an adhesive tape placed approximately 1 cm from the tip of the tail for 6 min and videotaped. Immobility was defined as the absence of any limb or body movements, apart from those required for respiration when the mouse was hung passively. The duration of immobility during the final 4 min of each test was recorded by an observer who was unaware of the grouping. During the test, the mice were separated from each other to prevent visual and acoustic associations.

###### Open Field Test

In this paradigm, the mice were placed at the center of an open field (50 ×50 × 40 cm) constructed of Plexiglas and allowed to explore the field for 5 min (Strekalova et al., 2004). The activity of each mouse was monitored using a video-tracking system (Shanghai Mobile Datum Information Technology Company, Shanghai, China) in the absence of an observer. The total distance and the number of rearing in the open field were measured. All animal behavior experiments were conducted at a light intensity of 20 lx.

#### Pharmacological Treatments

To observe the effects of the antidepressants, DD was used on both LH rats and CMS mice. The experimental protocol is shown in Figure 1A. After the LH model was established, rats were randomly divided into three groups (LH, LH + vehicle, and LH + DD groups). All DD groups were treated with DD (20 mg/kg/qd), whereas the vehicle group was administered the vehicle (mixture of anhydrous ethanol and olive oil in a 1:9 ratio) and the model group did not receive any treatment. Moreover, the drug and vehicle were administered to LH rats at 9:00 a.m. by gavage for 5 days. The CMS mice were randomly divided into three groups (CMS, CMS + vehicle, and CMS + DD groups). The experimental protocol is shown in Figure 1B. From the 6th week of CMS, the antidepressant group started to receive gavage of DD (20 mg/kg/qd) for 3 weeks. And behavioral tests were conducted once a week.

#### Hormone Assays

At the end of the experiment, all the rodents were rapidly decapitated (within 1 min after being taken from their home cage) using a guillotine between 10:00 and 12:00 h (i.e., 5–7 h after lights-on). Blood samples were taken from the hearts and collected in heparinized anticoagulant tubes for serum extraction. Serum levels of ACTH and cortisol were determined using a commercial enzyme linked immunosorbent assay kit (ACTH: Phoenix Pharmaceuticals Inc.; Cortisol: Demeditec Inc.) following the manufacturer’s instructions. If the levels of hormones in the samples were beyond the detection limit of the kits, the samples were diluted proportionally and tested again. The concentration of the original sample was then calculated by conversion. The kit specifications for each hormone are as follows (Table 1).

**Table 1:** Kit specifications for each hormone.

Hormone	Manufacturer	Specimen	Detection limit
ACTH	Phoenix Pharmaceuticals	serum	0.04 ng/ml
Cortisol	Demeditec Diagnostics	serum	0.1 ng/ml

*Abbreviations: ACTH, adrenocorticotropic hormone. Optical density was mostly measured at 450 nm using Epoch multi-volume spectrophotometer system (Bio-Tek Instruments Inc., USA) with the exception that the A/NA kit was used at 405 nm. Hormone concentrations were then calculated using Gen5 software (Bio-Tek Instruments)*.

#### Plasma Cytokine Analysis

In this study, plasma samples were used for IL-2, IL-4, IL-6, IL-10, and tumor necrosis factor (TNF)-α using the Bio-Plex ProTM Kit (Bio-Rad Laboratories, Hercules, CA, USA) according to the manufacturer’s instructions. Finally, the Bio-Plex 200 system and Bio-Plex Manager software (Bio-Rad Laboratories) were used to analyze the results.

#### Statistical Analysis

Data are expressed as the mean ± SEM and analyzed using SPSS 21.0. Data were tested for normality of distribution using the Kolmogorov Smirnov test, whereas homogeneity of variances was evaluated using Levene’s test. All the behavioral data were analyzed by two-way ANOVA, and only the inflammatory factor data were analyzed by one-way ANOVA. Duncan’s multiple range test was used for *post hoc* comparisons between the groups. *P* < 0.05 was considered statistically significant.

## Results

### Sub-chronic Administration of Daidzein Was Sufficient to Alleviate Inescapable Shock-Induced Helpless Behavior in LH Rats

**Figure 2:**
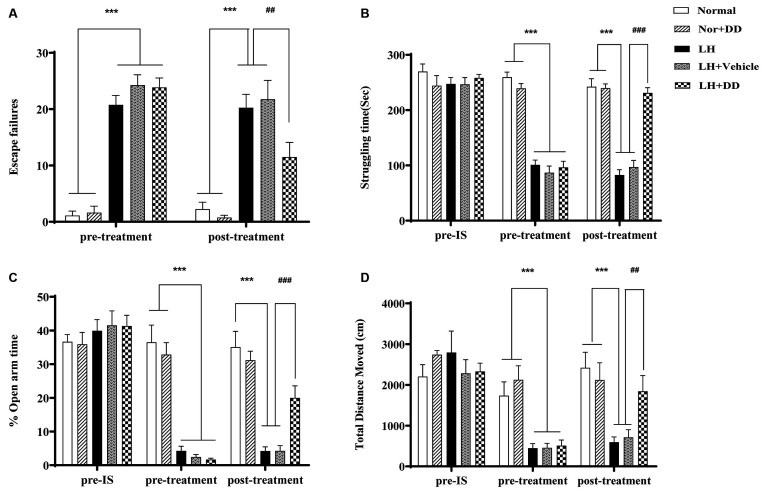
Sub-chronic administration of daidzein was sufficient to alleviate inescapable shock- induced helpless behaviors in LH rats. **(A)** Inescapable shock significantly increased the number of escape failures during the shuttle-box test. Daidzein alleviated an inescapable shock-induced deficit in escape behaviors. **(B)** After being exposed to inescapable shock, helpless rats showed even less struggle compared with the normal group, which could be improved by the daidzein treatment. **(C)** The percentage of the duration spent on the open arms of the elevated plus-maze was more significantly reduced after inescapable shock in the helpless rats compared with the normal group rats, similarly, which could be ameliorated by the daidzein treatment. **(D)** The total distance rats moved in the open field test (OFT) was also significantly decreased in the helpless rats compared with the normal group rats, in the same way, which could be perfected by the daidzein treatment. ***p < 0.001, ^###^*p* < 0.001, ^##^*p* < 0.01.

As shown in Figure 2A, the increased number of escape failures in the shuttle-box test signified that inescapable shock significantly impaired the escape behavior of rats (*F* = 69.284, *p* < 0.001), which confirmed the success of establishing the LH paradigm. After administration of DD, the inescapable shock-induced deficit in escape behavior was significantly alleviated (*p* < 0.01 vs. LH model). Similarly, the struggling time in FST following sub-chronic DD treatment on helpless rats is shown in Figure 2B. After exposure to inescapable shock, the helpless rats showed a decreased struggle (an index of depression-like state; *F* = 35.767, *p* < 0.001). Two-way ANOVA revealed that DD significantly increased the struggling time compared to the LH group (*p* < 0.001 vs. model). In the elevated plus-maze test, the LH rats spent significantly less time in the open arms, which was consistent with an “anxiety-like” behavior (Figure 2C; *F* = 43.156, *p* < 0.001 vs. normal). However, after administration of DD, the time the helpless rats spent in the open arms increased significantly (*p* < 0.001 vs. model), which was not observed in the helpless rats treated with vehicle. Similarly, in the OFT, the total distance covered by the helpless rats also significantly decreased (Figure 2D; *F* = 29.375; p < 0.001 vs. normal), whereas DD significantly reversed this depression-like behavior by increasing the total distance (*p* < 0.01 vs. model). However, DD had no obvious effect on the behavior of normal rats (*p* > 0.05, normal + DD group vs. normal group).

### Chronic Administration of Daidzein Relieved Depression-Like Behaviors in CMS Mice

**Figure 3:**
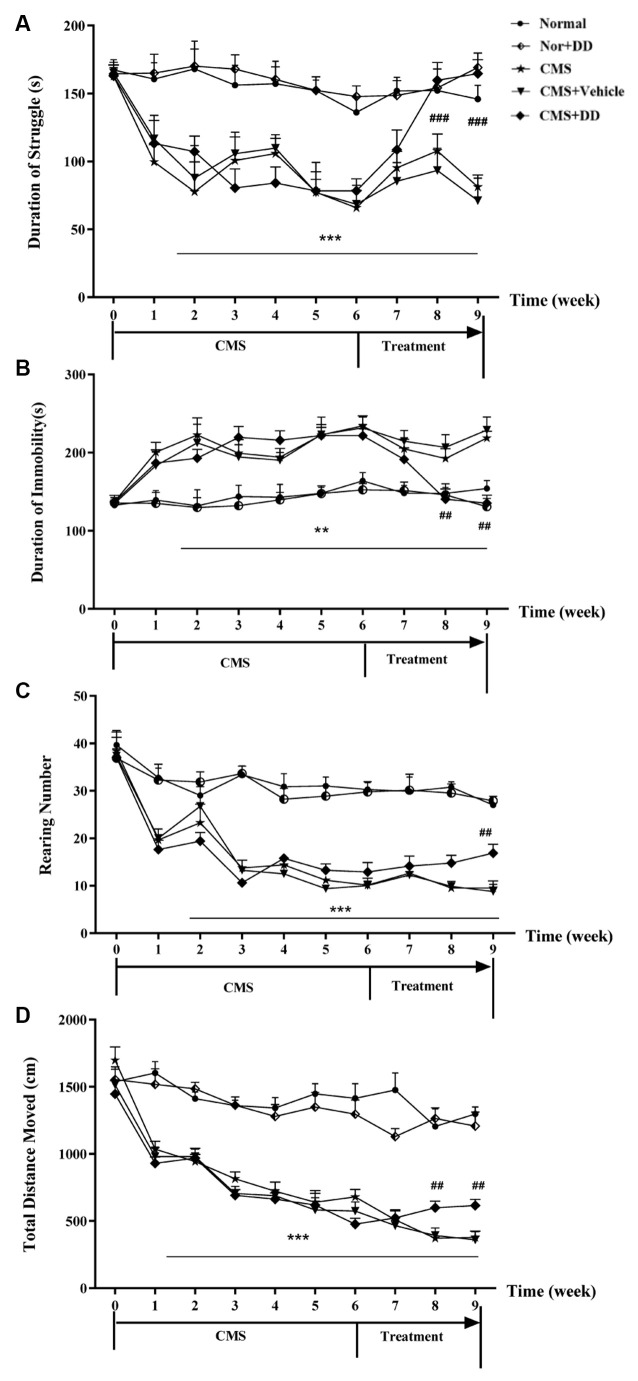
Chronic administration of daidzein relieved depression-like behaviors in CMS mice. **(A)** CMS mice spent significantly less time struggling in the forced swimming test (FST) compared to normal mice, while daidzein contradicted the effect of CMS on the despair behavior compared to CMS mice. **(B)** Daidzein showed an antidepressant effect on CMS mice in tail suspension test (TST). **(C)** Chronic mild stress (CMS) induced impaired explorative behavior and the decreased rearing number in OFT compared to normal mice which was relieved by DD. **(D)** The total moving distance of the rats in the open field test was also significantly decreased in CMS mice compared with that of the normal group, which suggested improvement brought about by daidzein treatment. Whereas, DD displayed no obvious effect on the behavior of normal rats. **p < 0.01, ***p < 0.001, ^##^*p* < 0.01, ^###^*p* < 0.001.

As illustrated in Figure 3, the mice subjected to CMS exhibited long-lasting depression-like behaviors for 6 weeks, since a similar pattern of behavioral deficits was found in the weeks after the onset of stress, and as the treatment progressed, DD significantly reversed the depression-like behavior induced by CMS.

Chronic mild stress induced depression-like behavior, which was represented by less struggling time in the TST (Figure 3A; *F* = 41.593, *p* < 0.001 vs. normal group), significantly increased immobility period in the FST (Figure 3B; *F* = 5.913, *p* < 0.01 vs. normal group), decreased rearing number and shortened total moving distance in the OFT (Figures 3C,D; *p* < 0.001 vs. normal group). However, DD relieved these depression-like behaviors, which was represented by longer struggling time in the TST (*p* < 0.001, DD vs. CMS model group), decreased immobility period in the FST, and increased rearing number and the total distance in the OFT (*p* < 0.01, DD vs. CMS model group). However, DD had no obvious effect on the behavior of normal rats (*p* > 0.05, normal + DD group vs. normal group).

### Chronic or Sub-chronic Administration of Daidzein Restored Stress-Induced Disturbances in the HPA Axis

**Figure 4:**
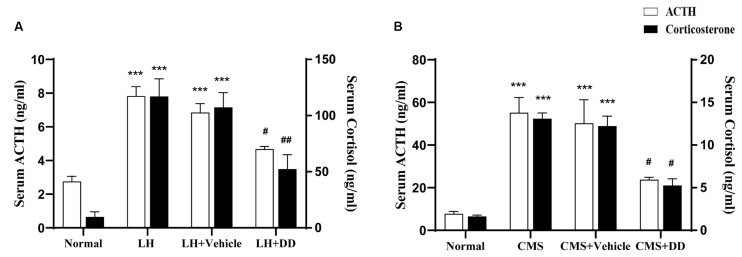
Sub-chronic and Chronic administration of daidzein restored stress-induced disturbances in the hypothalamic pituitary adrenal (HPA) axis. **(A)** Sub-chronic administration of daidzein showed that there was significant increase in ACTH levels after rats were submitted to inescapable shock as compared to the normal group. Sub-chronic daidzein decreased serum ACTH levels compared to the LH group. Application of inescapable shock also increased corticosterone as compared to the normal group. Daidzein decreased serum corticosterone level significantly compared with the LH group. No difference was found in serum corticosterone levels between the LH group and vehicle group (*p* > 0.05). **(B)** Serum levels of ACTH and corticosterone were significantly higher in CMS mice compared to normal group. Daidzein administration for 3 weeks, significantly attenuated the increase of serum ACTH and corticosterone in CMS mice. ***p < 0.001, ^#^*p* < 0.05, ^##^*p* < 0.01.

The LH group displayed increased levels of ACTH (Figure 4A; *F* = 8.269, *p* < 0.001 vs. normal group) and corticosterone (Figure 4A; *F* = 13.683 *p* < 0.001 vs. normal group), whereas sub-chronic administration of DD decreased the levels of both (ACTH: *p* < 0.05 vs. model group) (corticosterone: *p* < 0.01 vs. model group). No difference was found in the serum corticosterone levels between the model and saline groups (*p* > 0.05).

In line with the previous results in LH rats, serum levels of ACTH and corticosterone were significantly higher in CMS mice than in non-CMS controls (Figure 4B; ACTH: *F* = 12.378, *p* < 0.001; corticosterone: *F* = 11.951, *p* < 0.001). Compared with the model group, the DD group displayed an attenuated increase in ACTH and corticosterone levels in the serum (Figure 4B; ACTH: *p* < 0.05 vs. Model; corticosterone: *p* < 0.05, vs. model group). Similarly, we did not observe any adverse effects of DD on normal mice (*p* > 0.05 vs. normal group).

### Chronic or Sub-chronic Daidzein Treatment Rectified Stress-Induced Imbalances of Circulating Cytokines

**Figure 5:**
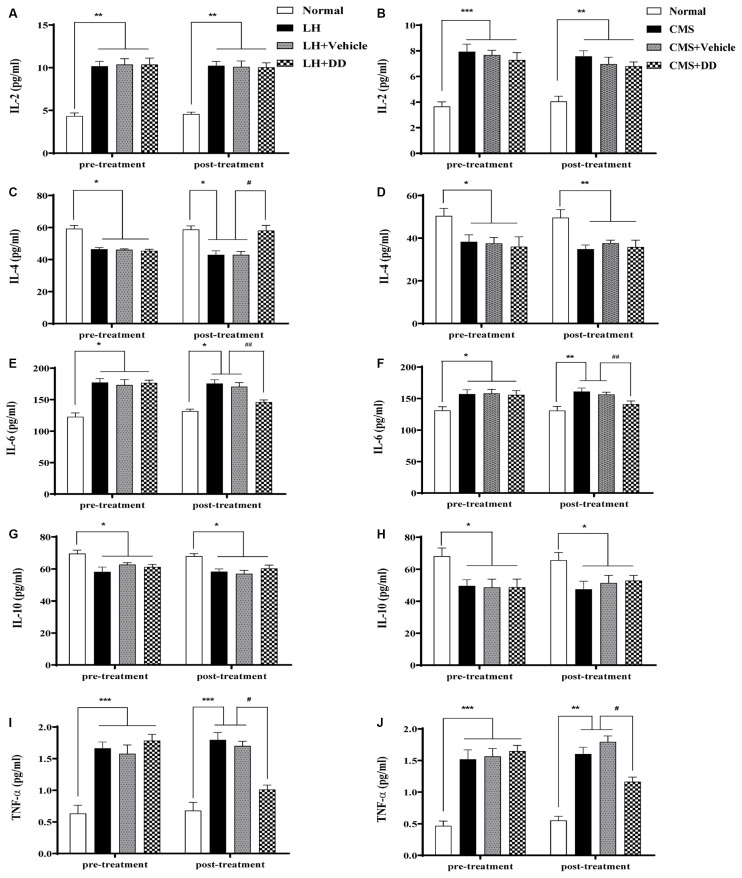
Sub-chronic or chronic daidzein treatment rectified stress-induced imbalances of circulating cytokines. **(A,C,E,G,I)** LH model produced a significant imbalance in serum circulating cytokines, which involved upregulation of interleukin (IL)-2 and IL-6, and downregulation of IL-4, IL-10, and tumor necrosis factor (TNF)-α compared with the normal group. After sub-chronic treatment with DD, IL-4 was significantly increased, while IL-6 and TNF-α were also significantly reduced compared with the LH level, whereas no significant change in IL-2 and IL-10 levels was observed compared with the LH model rats. **(B,D,F,H,J)** Likely, CMS produced significant upregulation of IL-2 and IL-6, and downregulation of IL-4, IL-10, and TNF-α compared with the normal group. After chronic treatment with DD, IL-6 and TNF-α were significantly reduced compared with the CMS group, no significant change in IL-2 level was observed; IL-4 and IL-10 were slightly increased towards the normal level compared with CMS group. *p < 0.05, **p < 0.01, ***p < 0.01, ^#^*p* < 0.05, ^##^*p* < 0.01.

To assess the effects of chronic stress and chronic DD treatment on circulating cytokines, the serum levels of IL-2, IL-4, IL-6, IL-10, and TNF-α were assessed (Figure 5). It was found that CMS produced a significant imbalance in serum circulating cytokines, which involved upregulation of IL-2 and IL-6, and downregulation of IL-4, IL-10, and TNF-α (Figures 5B–J, IL-2: *p* < 0.001; IL-4: *p* < 0.05; IL-6: *p* < 0.05; IL-10: *p* < 0.05; TNF-α: *p* < 0.001 vs. normal group).However, after chronic treatment with DD, the following alterations were observed: IL-6 and TNF-α were significantly reduced, no significant change in IL-2 level was observed (IL-2: *p* > 0.05; IL-6: *p* < 0.01; TNF-α: *p* < 0.001 vs. model group); IL-4, and IL-10 were slightly increased towards the normal level (IL-4: *p* > 0.05; IL-10: *p* > 0.05 vs. model group). The same inflammatory circulating cytokines were also detected to evaluate acute stress and rapid DD treatment in LH rats. Similarly, the LH model produced a significant imbalance in serum circulating cytokines, which involved upregulation of IL-2 and IL-6, and downregulation of IL-4, IL-10, and TNF-α (Figures 5A–I, IL-2: *p* < 0.01; IL-4: *p* < 0.05; IL-6: *p* < 0.05; IL-10: *p* < 0.05; TNF-α: *p* < 0.001 vs. normal group). After treatment with DD, the following alterations were observed: IL-4 was significantly increased towards the normal level (*p* < 0.05), while IL-6, and TNF-α were also significantly reduced compared with the model level (*p* < 0.05), whereas no significant change in IL-2 and IL-10 levels was observed compared with the LH model rats (*p* > 0.05).

## Discussion

As mentioned above, anxiety disorders are closely related to estrogen and its receptors. Studies suggest that estrogen deficiency results in NLRP3 inflammasome activation, leading to neuroinflammation in the hippocampus and depression and anxiety. Estrogen modulation of inflammation in the hippocampus and depression-and anxiety-like behaviors are estrogen receptor β (Erβ)-dependent (Xu et al., 2016). Meanwhile, ERβ can inhibit HPA reactivity and decrease anxiety-like behavior in rodents (Kudwa et al., 2014). The phytestrogen DD and its metabolites can combine with estrogen receptors to play a related biological role.

Rationale Serotonin (5-HT) neurotransmission is intimately linked to anxiety and depression (Garcia-Garcia et al., 2014). There is even a famous hypothesis of “low serotonin function”. The researchers believe that low brain 5-HT exacerbates depression-and anxiety-like behavior induced by stress and blocks reductions in depression-like behavior induced by antidepressants (Karth et al., 2019). Since less than one-third of patients with major depression attain remission with an antidepressant trial, which indicates that there may be other mechanisms involved in the occurrence of anxiety and depression. Just as we mentioned above, inflammation or HPA axis activation may also be involved in the pathogenesis of anxiety disorder. We speculate that DD may play an anti-anxiety role by regulating the HPA axis and inflammatory response.

Stress is considered a trigger factor for many affective disorders, including major depression. Stress-based animal models represent a useful instrument to mimic depressive symptomatology (Slattery and Cryan, 2017), such as anhedonia, despair, and some other symptoms. In addition to depressive-like behavior, inescapable shock or chronic mild stress in rodents also induces hyperactivity of the HPA axis and imbalance of inflammatory factors (Farooq et al., 2018; Ludwig et al., 2019). Some neuroendocrine changes observed in the LH or CMS-induced depression model were similar to those observed in patients with depression (Edwards et al., 2000; Grippo et al., 2005). To test whether DD had a rapid and stable anti-anxiety effect, two rodent models of depression (LH and CMS models) were used to verify whether the antidepressant effects of daidzein were associated with the rectification of HPA axis hyperactivity (represented by increased serum levels of ACTH and corticosterone) and the imbalance between pro- and anti-inflammatory cytokines. Our results showed that sub-chronic inescapable shock or CMS induced depression-like behaviors in rodents, accompanied by higher serum levels of ACTH and corticosterone and an imbalance of circulatory cytokines represented by an upregulation of pro-inflammatory cytokines and downregulation of anti-inflammatory cytokines. In the current study, our results displayed the positive anti-anxiety effect of daidzein, which improved the depression-like behaviors of the two models. The increased ACTH and corticosterone levels were significantly reversed by sub-chronic or chronic treatment with DD, which was accompanied by significant rectification in the imbalance between much of pro- and anti-inflammatory cytokines.

There have been two distinctive hypotheses regarding the cause of depression: hyperactivity of the HPA axis and imbalance between pro- and anti-inflammatory cytokines. The HPA axis has been considered a potential fast-acting functional system related to behavioral symptoms observed in acute or sub-chronic experimental stress paradigms (Crowley and Girdler, 2014). In addition, the major role of the HPA axis in stress-related conditions has rendered it as a marker for stress responses and as a mediator for the subsequent downstream long-lasting pathophysiological changes (Mello et al., 2003). Hence, disruption of the HPA axis is thought to be involved in the pathophysiology of mood and anxiety disorders (Min et al., 2012; Zhao et al., 2018). In accordance with this assumption, our study showed that when exposed to sub-chronic stress or chronic stress, rodents exhibited greater ACTH or corticosterone levels than the controls. Alternatively, researchers have been focusing on the relationship between stress, immune system, and depression since the onset of this century, and different results have been obtained. A parallel meta-analysis revealed that several pro-inflammatory cytokines, including IL-1, IL-6, and TNF-α, were related to the development of depression (Coplan et al., 2014), and increased levels of pro-inflammatory cytokines, such as IL-6, C-reactive protein, and TNF-α, were also detected in the cerebrospinal fluid and serum of depressed individuals, who were otherwise healthy. However, some studies failed to find a relationship between pro-inflammatory cytokines and depression, which was assumed to be confounded by other factors, such as sex, body mass index, or personality. An inverse correlation between these two has also been confirmed (Raison et al., 2006). It is worth noting that most of the studies mentioned above have focused only on pro-inflammatory cytokines while ignoring anti-inflammatory cytokines. Dantzer and Kelley found that the sickness behavior caused by IL-1β and TNF-α was regulated *via* inhibition of their production and signaling by anti-inflammatory cytokines, such as IL-10. Therefore, he assumed that the development of depression may be related to both pro- and anti-inflammatory cytokines and argued for the critical role of the equilibrium between pro- and anti-inflammatory cytokines in the maintenance of non-depressive states (Dantzer and Kelley, 2007). Based on this research, we selected IL-10 and some other well-known pro- and anti-inflammatory cytokines in our study to investigate the relationship between the immune response and the development of depression. Our results suggest that chronic stress may induce depression-like behavior by upsetting the balance between pro-inflammatory and anti-inflammatory cytokines. Furthermore, although chronic treatment with DD alleviated depression-like behavior, it failed to rectify the imbalance completely because it only changed IL-6 and TNF-α levels in the serum of both anxiety models, which implied that the imbalance between pro- and anti-inflammatory cytokines was not entirely consistent with depression symptoms. IL-6 and TNF-α may play a more important role than other cytokines in the pathogenesis of depression. The inconsistencies in the relationship between depression and inflammatory cytokines incurred challenged the hypothesized “causal” role of cytokines in depression, which led to the argument that hyperactivity of the HPA axis is the “crucial” link between psychosocial stress and depression. Hence, the question is whether hyperactivity of the HPA axis and the imbalance between cytokines are more predominant in determining the occurrence of depression. Although our results failed to confirm the causal relationship between them, on the basis of previous studies that showed that hormones secreted from the HPA axis increased pro-inflammatory cytokines, we suggest that CMS triggers depression-like behavior *via* the effects of hormones secreted from the HPA axis, such as corticotropin-releasing factor, ACTH, or glucocorticoids, which upset the equilibrium of circulatory cytokines in an animal model of depression. Moreover, DD alleviated the depression-like behavior and normalized activity of the HPA axis but failed to rectify the imbalance of all cytokines. Therefore, we presume that the primary causal role for depression should be the increased HPA hyperactivity-derived hormones, which, in turn, lead to elevated pro-inflammatory responses; therefore, the dysregulation of the HPA axis appears to be more closely related to depression.

Meanwhile, it is also important to note that the high levels of ACTH and corticosterone were decreased in the LH rats and CMS mice after chronic and sub-chronic treatment with DD, which was consistent with the alleviation of depression-like behavior. Inferred from the above, it is promising that pharmacological intervention may relieve depression behavior by reducing stress hormone levels. One possible explanation is that antidepressant administration partially desensitizes the HPA axis through the post-synaptic 5-HT receptors in the paraventricular nucleus (PVN) of the hypothalamus, which then results in decreased ACTH release at the pituitary level (Franklin et al., 2013; Otsuka et al., 2016). In addition, studies have already demonstrated that HPA activation is associated with a 5-HT1A agonistic function by increasing plasma corticosterone levels to help the animal cope with stressful conditions (Pineda et al., 2011; Gupta et al., 2013). Thus, it is evident that the changes in the levels of ACTH and corticosterone caused by DD in the present study were in line with these previous observations.

In conclusion, our findings confirmed the roles of the hyperactive HPA axis and abnormal circulatory cytokines in rodent models of depression and demonstrated the effects of daidzein in attenuating depression-like and anxiety-like behaviors with decreased stress hormone levels and unrectified imbalance of cytokines. However, there is a deficiency that the effect of DD on the anxiety model in female rats was not confirmed. Further study is needed in the follow-up experiments.

## Data Availability Statement

The original contributions presented in the study are included in the article, further inquiries can be directed to the corresponding author/s.

## Ethics Statement

The animal study was reviewed and approved by the Ethics Committee of Animal Care of Jinshan Hospital.

## Author Contributions

BL and PG designed and drafted the manuscript. LC, YZ, and XW performed the experiments. HZ and CW analyzed the results. All authors contributed to the article and approved the submitted version.

## Conflict of Interest

The authors declare that the research was conducted in the absence of any commercial or financial relationships that could be construed as a potential conflict of interest.

## Publisher’s Note

All claims expressed in this article are solely those of the authors and do not necessarily represent those of their affiliated organizations, or those of the publisher, the editors and the reviewers. Any product that may be evaluated in this article, or claim that may be made by its manufacturer, is not guaranteed or endorsed by the publisher.
